# Delphi Analysis: Optimizing Anatomy Teaching and Ultrasound Training for Botulinum Neurotoxin Type A Injection in Spasticity and Dystonia

**DOI:** 10.3390/toxins16080371

**Published:** 2024-08-21

**Authors:** Kimberly Heckert, Bo Biering-Sørensen, Tobias Bäumer, Omar Khan, Fernando Pagan, Mitchell Paulin, Todd Stitik, Monica Verduzco-Gutierrez, Rajiv Reebye

**Affiliations:** 1 Department of Rehabilitation Medicine, Thomas Jefferson University, Philadelphia, PA 19107, USA; 2 Department of Neurology, Rigshospitalet, Glostrup, DK-2600 Copenhagen, Denmark; 3 Institute of System Motor Science, Center of Brain, Behavior and Metabolism, University of Lübeck, 23562 Lübeck, Germany; tobias.baeumer@neuro.uni-luebeck.de; 4 Hotel Dieu Shaver Health and Rehabilitation Centre, St. Catharines, ON L2T 4C2, Canada; odkpmr@gmail.com; 5 Department of Neurology, Georgetown University, Washington, DC 20007, USA; fernando.l.pagan@gunet.georgetown.edu; 6 Rehabilitation Associates of the Main Line, Main Line Health, Paoli, PA 19301, USA; titch@prodigy.net; 7 Department of Physical Medicine and Rehabilitation, Rutgers New Jersey Medical School, Newark, NJ 07103, USA; todd.stitik@gmail.com; 8 Rehabilitation Medicine, UT Health San Antonio, San Antonio, TX 78229, USA; gutierrezm19@uthscsa.edu; 9 Department of Medicine, University of British Columbia, Vancouver, BC V5Z 2G9, Canada

**Keywords:** botulinum neurotoxin, dystonia, movement disorder, spasticity, training programs, ultrasound imaging

## Abstract

Our objective was to provide expert consensus on best practices for anatomy teaching and training on ultrasound-guided botulinum neurotoxin type A (BoNT-A) injection for specialists involved in treating spasticity and dystonia. Nine experts (three neurologists; six physical medicine and rehabilitation physicians) participated in a three-round modified Delphi process. Over three rounds, experts reached consensus on 15 of 16 statements describing best practices for anatomy and BoNT-A injection training. They unanimously agreed that knowledge of the target audience, including their needs and current competency, is crucial when designing training programs. Experts also agreed that alignment between instructors is essential to ensure consistency of approach over time and between regions, and that training programs should be simple, adaptable, and “hands-on” to enhance engagement and learning. Consensus was also reached for several other key areas of training program development. The best-practice principles identified by expert consensus could aid in the development of effective, standardized programs for anatomy teaching and BoNT-A injection training for the purposes of treating spasticity and dystonia. This will enhance the exchange of knowledge, skills, and educational approaches between global experts, allowing more specialists to treat important movement disorders and ultimately improving patient outcomes.

## 1. Introduction

Botulinum neurotoxin type A (BoNT-A) is a well-established treatment for movement disorders characterized by muscle hyperactivity [1]. Indeed, based on Level A evidence from high-quality clinical trials, BoNT-A is recommended as first-line treatment for spasticity and cervical dystonia, as its use in these disorders improves outcomes and functional recovery [2,3].

As BoNT-A is injected directly into the muscles involved in the movement disorder, accurate muscle targeting is essential [4]. Precise localization ensures that BoNT-A reaches the right muscle and minimizes both the risks of the injection procedure and side effects from toxin spread [1,5]. As BoNT-A may induce muscle weakness, a side effect of BoNT-A with poor localization is weakness in unintended muscles [6], which may have functional consequences that defer patients from returning for additional treatments [7]. Needling of neurovascular structures or multiple needle passes to reach targets are likely to further defer patients from returning due to discomfort, post-injection bleeding/bruising, and pain [7]. Several injection guidance techniques are available to aid muscle localization in BoNT-A treatment, including manual palpation based on surface landmarks and instrumented guidance techniques, such as electromyography (EMG), electrical stimulation (e-stim), and ultrasound (US) [1,5]. The benefits of instrumented guidance methods (versus manual palpation alone) are now widely acknowledged and documented [3,8,9,10,11,12,13,14], and have recently been confirmed in a quantitative analysis [5].

Achieving effective treatment outcomes with BoNT-A in spasticity and dystonia therefore requires physicians to develop not only a detailed knowledge of muscle anatomy and surrounding structures [1,8,15,16], but also a comprehensive understanding of injection guidance techniques [4]. Accordingly, guidelines such as those from the Royal College of Physicians in the UK recommend that BoNT-A injections should be administered only by trained physicians with appropriate expertise [17]. However, to the best of our knowledge, there are currently no standardized, widely accepted best practices for how to effectively design training programs for BoNT-A injection or how to incorporate anatomy teaching into such programs. Therefore, the aim of this study was to provide expert consensus on best practices for anatomy and BoNT-A injection training, and to investigate whether cadaveric anatomy teaching has a place in these programs.

## 2. Results

### 2.1. Round 1

All nine experts attended the face-to-face advisory board meeting in Round 1. Based on discussions from this meeting, experts generated 16 primary statements regarding best practices for designing anatomy and BoNT-A injection training courses (Table S1). The statements addressed a variety of components for training programs, including objectives, eligibility criteria for both instructors and participants, curriculum design, engagement and assessment, and course content. Sub-statements were generated for six of the primary statements. The 16 statements were circulated to the expert panel for voting in Round 2.

### 2.2. Round 2

Eight of the nine experts participated in Round 2 voting. Experts reached unanimous agreement (100% consensus) on 11 of 16 (69%) primary statements (statements 1–6, 10, and 12–15); consensus was also reached for eight of 12 (67%) sub-statements (sub-statements 1a, 1b, 2b, 5a, 6a, 9a, 9b, and 16a), at agreement levels of 88–100%. Five of 16 (31%) primary statements (statements 7–9, 11, and 16) and 4 of 12 (33%) sub-statements (sub-statements 2a, 2c, 5b, and 16b) failed to achieve consensus (25–75% consensus) (Table S2).

All statements that reached consensus were added to the list of final statements (Table 1), while those that failed to reach consensus were modified according to the experts’ feedback and sent out for re-evaluation in the Round 3 questionnaire.

### 2.3. Round 3

All nine experts participated in Round 3. In this round, experts reached consensus on four of the five primary statements and all four sub-statements that did not reach consensus in Round 2 (all at a level of 88–100% agreement). Therefore, by the end of Round 3, 15/16 (94%) primary statements and 12/12 (100%) sub-statements had achieved consensus (Table 1).

### 2.4. Consensus Recommendations

Finalized statements and consensus levels are presented in Table 1. Experts unanimously agreed (100%) that the core learning objectives of a training program for anatomy and BoNT-A injection techniques should be tailored to the target audience and region by considering how existing educational programs could be enhanced and the audience’s knowledge and competency levels improved. As such, experts also agreed that the target audience is a crucial component of program design (100%) and that course participants may need to meet certain qualifications or be recommended for inclusion (100%). Depending on the local scope of practice, most experts felt that physiotherapists and occupational therapists should be eligible to participate in training programs to enhance their knowledge of anatomy related to post-stroke spasticity (89%), as this group often refers patients to specialists for treatment (including BoNT-A injection). However, it is widely acknowledged that in many countries, allied health professionals are legally restricted from administering BoNT-A injections to patients, in accordance with local regulations. Experts discussed that while there may be benefits to allowing nurses, physiotherapists, and occupational therapists to administer BoNT-A injections, the ultimate responsibility must always rest with the consulting physician (neurologist or physical medicine and rehabilitation [PM&R] specialist), as mandated by law.

There was widespread agreement that an optimal training curriculum should be simple and adaptable (100%), that it should impart skills that can be assessed objectively (100%), and that it would ideally incorporate hands-on activities to facilitate and demonstrate engagement (100%), including the opportunity to use injection-guidance techniques (100%). Experts agreed that training programs should remain uniform over time and between different regions, with consistent content and use of nomenclature (100%), and that trainers should be selected on the basis of competency using principles of inclusion and diversity (89%).

When considering the level of prior knowledge of US required for participation in training programs, experts broadly agreed that participants should understand the foundational principles (such as knobology and ergonomics) before attending the training (89%). This background knowledge could be acquired through the participants’ educational or clinical backgrounds or through pre-training educational materials. Experts also agreed that the benefits and limitations of other injection-guidance techniques, such as EMG and e-stim, should be discussed in programs that focus on US (88%). There was unanimous agreement (100%) that BoNT-A injections should be considered an important part of a broader, holistic, multimodal treatment approach. Similarly, unanimous consensus (100%) was also reached for several additional recommendations: Namely, training programs should offer instruction on treatment goal-setting and patient evaluation, “train the trainer” meetings should be used to ensure alignment between instructors, training programs would benefit from leveraging strengths in local university or hospital departments, and program participants should have an opportunity to assess their instructors.

All experts agreed that, when available, cadavers (whole-body or prosected) are the optimal specimens to enhance anatomy teaching for the purposes of US-guided BoNT-A injection in spasticity and dystonia (100%). Virtual models might be an appropriate alternative, to some extent, if no cadaveric specimens or prosections are available (88%). Artificial models are additional tools for teaching US and other injection-guidance techniques alongside cadavers (100%).

Just one statement (“As part of a holistic multimodal approach to treat movement disorders, the word ‘injector’ should be replaced with ‘experts in the management of spasticity and movement disorders’”) failed to reach consensus after Round 3.

## 3. Discussion

Currently, several programs offer training in anatomy and BoNT-A injection techniques, such as the international Ixcellence Network^®^ [18,19]; the European Musculoskeletal Ultrasound Study Group (EURO-MUSCULUS)/Ultrasound Study Group in Physical and Rehabilitation Medicine (USPRM) program [20]; the Scandinavian Diploma Education in Dystonia and Spasticity Treatment (SKANDYSPAS) program in Denmark, Norway, and Sweden [21]; country-specific training programs in Germany [22] and France [23]; society-specific training programs (such as the STEP Interventional Spasticity Certificate Program from the American Academy of Physical Medicine and Rehabilitation [24]); and a recently piloted, inter-institutional, hybrid training curriculum for PM&R residents in the USA [25]. However, whether training programs are tailored towards physicians in university and specialty education settings or towards neurologists and PM&R specialists in clinical practice, there are currently no standardized guidelines for curriculum design to ensure that the programs are adequate. We believe the current research therefore addresses a significant gap, and we hope that our best-practice recommendations will enhance and standardize existing training programs and guide the development of such programs in countries where they are currently lacking.

Experts unanimously affirmed the crucial importance of considering the target audience. Taking into account the participants’ knowledge and skill levels will help trainers and faculty to tailor educational activities to the audience’s learning needs, thus maximizing immediate benefits and instilling confidence in the participants to put their skills into practice.

**Figure 1:**
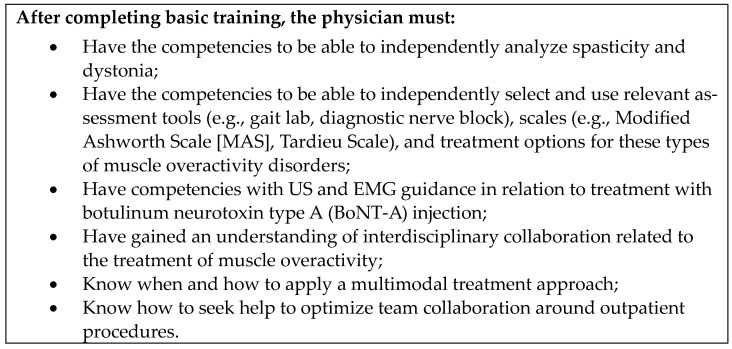
Example goals for a simple curriculum design ^1^ for anatomy and BoNT-A injection training courses. ^1^ Based on the Scandinavian Diploma Education in Dystonia and Spasticity Treatment (SKANDYSPAS) training program [21].

Unanimous agreement was also reached concerning the need for instructors to align on training techniques, nomenclature, and messaging; this could be facilitated by a pre-training faculty meeting. Experts also agreed that training programs should be as simple as possible, using only what most physicians would typically have in their office (such as a US machine, a needle, and a “patient”). Simplicity in terms of curriculum content ensures that training goals are clear, succinct, and easily assessed (for an example, see Figure 1). Where participants require comprehensive information about assessment and/or management of specific movement disorders, pre-existing educational materials may be used, such as the Toxnet curriculum. Toxnet is a global education initiative that aims to raise awareness of best clinical practices in spasticity and offers a comprehensive “blueprint” training course [10,15,26,27,28]. Training programs should also be adaptable so they can accommodate country-specific needs (e.g., product labeling) and audience characteristics, such as skill level. While learners can be stratified prior to training, levels of skill and understanding may be different than anticipated, and in such cases the trainer would need to adapt training to the learners’ needs.

Another core principle of effective training programs is maximization of engagement and learning by prioritizing hands-on, practical training over didactic sessions and by incorporating gamification techniques [29].

Substantial evidence and experience attests to the superiority of instrumented guidance versus manual palpation alone [1,5,14,30,31,32]. Accordingly, experts agreed that instructors should explain that manual palpation alone is not preferred for targeting muscles for BoNT-A injection, and the sole use of manual palpation should be considered only when other guidance methods are unavailable [11]. Most experts felt that course participants should be trained to use other techniques (e.g., EMG, e-stim) in programs that focus on US so that they become equipped to treat patients regardless of the available resources [5].

There was full agreement that, when available, cadavers are the optimal specimens for instruction on anatomy and US-guided injection (Table 2). However, prosected limbs could be used instead of whole-body cadavers and extensive dissection to save both time and money. Live models might also be helpful for demonstrating US guidance on real-life anatomy and for allowing participants to practice using EMG, which cannot be used on cadaveric specimens.

One statement failed to achieve consensus (“As part of a holistic multimodal approach to treat movement disorders, the word ‘injector’ should be replaced with ‘experts in the management of spasticity and movement disorders’”). While most panel members agreed that “injector” neither represents the skill and expertise necessary to administer BoNT-A injections nor recognizes that BoNT-A is part of a broader treatment approach, one expert did not agree with the suggested replacement term and another felt that “injector” was appropriate.

As a consensus approach, Delphi methodology is particularly useful in areas with limited empirical evidence, as it can form initial opinion and set the direction for scientific research [33]. One advantage of the method is anonymity [34,35]. In our modified Delphi, anonymity was implemented during Rounds 2 and 3, but not in Round 1. However, it is common for modified Delphi studies to include expert interaction, often as the first stage in the process [33,36] and typically taking the form of meetings and group discussions [37,38,39], advisory boards [40], or workshops [41]. In such cases, the benefits of face-to-face interaction, such as the ability to clarify reasons for disagreements, compensate for the lack of anonymity [36].

While our Delphi panel comprised only nine experts, all have extensive teaching and clinical experience and are representative of academic physiatrists involved in teaching BoNT-A injection techniques using US guidance. Several panelists have been authors of expert opinion papers in this area [3,42], and all use injection-guidance techniques in their clinical practice. Further, a Delphi panel of five to 10 participants is generally considered adequate [43,44].

One potential limitation of our study is that expert panel members only represented the USA, Canada, and Europe; unrepresented regions may have different educational needs and resources. Heterogeneous panels are generally recommended to obtain a wider range of perspectives with global relevance. This limitation could be overcome by inviting experts from other regions to repeat Round 2 and 3 questionnaires with subsequent analysis of aggregate data. Additionally, one expert was unable to participate in Round 2. However, this panel member reviewed the anonymized voting results from Round 2, agreed with the outcome, and went on to complete Round 3. Including panel members who missed a previous round in subsequent rounds of a Delphi process may reduce the risk of false consensus and enhance representation of the original panel’s opinions, without affecting the final outcome [45]. A further potential limitation was that no empirical validation of the findings of the Delphi process was performed to evaluate their effectiveness in improving clinical outcomes. Future work could aim to gather real-world evidence to ensure the effectiveness and applicability of these best-practice principles across a variety of healthcare settings, and expand on technical aspects to cover in the training programs.

## 4. Conclusions

In conclusion, and to the best of our knowledge, this is the first time a modified Delphi approach has been used to establish best practices for anatomy teaching and BoNT-A injection training for physicians who treat spasticity and dystonia. Knowledge of the target audience, instructor alignment, and use of a simple, adaptable curriculum were identified as core principles in designing effective training. It is hoped that the best-practice principles identified by this process will be used to develop effective, standardized training programs, with the goal of enhancing the exchange of knowledge, skills, and educational approaches between experts around the world, allowing more physicians to treat important conditions such as spasticity or dystonia, and ultimately improving patient outcomes.

## 5. Materials and Methods

### 5.1. Study Design

A three-round modified Delphi process (November 2022–April 2023) was used to build expert consensus on optimal approaches for training on anatomy and US guidance for BoNT-A injections in spasticity and dystonia. The Delphi technique is a widely accepted method for establishing consensus among experts on a specific issue or problem, especially where published evidence is limited or contradictory [43,46,47]. In this iterative process, experts rank their agreement with a series of statements in several rounds of voting until consensus is reached; while up to four rounds of voting may be used, two are usually sufficient [48].

**Figure 2:**
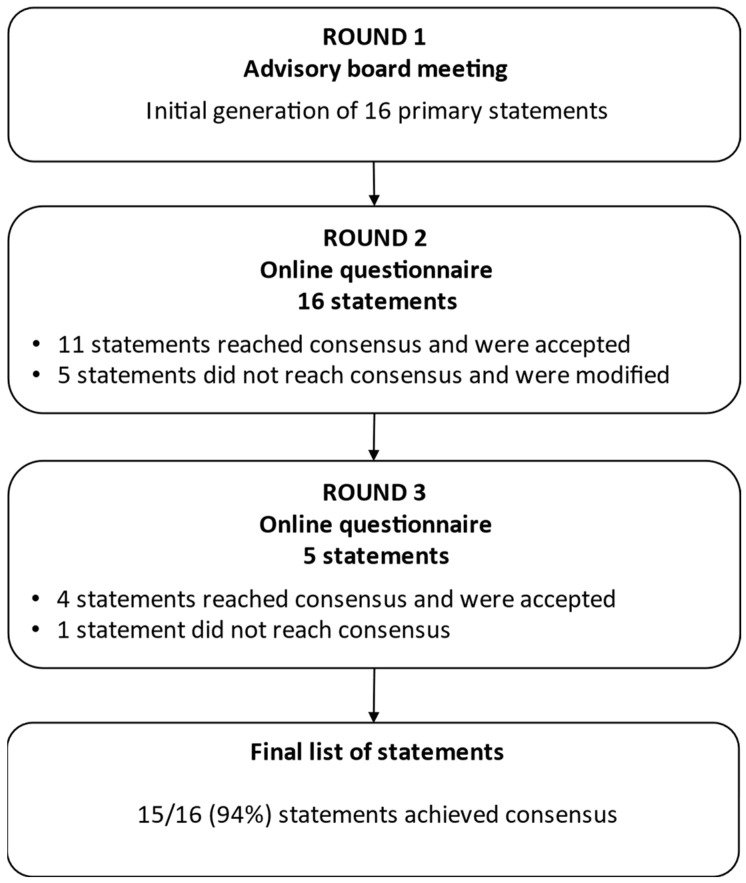
Modified Delphi process.

In the basic Delphi technique, experts provide feedback anonymously via self-administered questionnaires in all voting rounds [36]. In a modified Delphi process, however, self-administered questionnaires are typically combined with a face-to-face meeting to facilitate expert interaction [36,43], usually in the first round [33]. We used a modified Delphi process comprising one face-to-face meeting and two subsequent rounds of self-administered questionnaires (Figure 2).

### 5.2. Participants

Experts were selected on the basis of their academic background and experience in using BoNT. One major criterion was their involvement in designing and managing spasticity training programs, such as university, local, or national curricula. Given that more than half of the expert panel had extensive knowledge of dystonia and that best-practice principles for training program design would likely apply to both spasticity and dystonia, dystonia was also considered in the modified Delphi process. Another criterion was that each expert should have >10 years of experience with injecting BoNT and should be familiar with all available injection-guidance techniques, as well as still treating patients. Experts were selected from North America and Europe, as healthcare professionals in these regions have, on average, the greatest experience in treating spasticity with BoNT. The size of the expert panel was limited to allow constructive discussions in Round 1.

The expert panel was composed of nine healthcare professionals, comprising three neurologists and six PM&R physicians from the USA (n = 5), Canada (n = 2), Denmark (n = 1), and Germany (n = 1). These experts have a wealth of professional knowledge and experience in treating spasticity and dystonia, with >187 years of combined experience in using BoNT injection to treat these and other movement disorders. All panelists have academic appointments at universities and teaching hospitals, and all serve as faculty members, chairs, or co-chairs on national or international teaching programs on anatomy and/or the use of US. Example expert opinion publications from panelists include an article evaluating the ergonomics of US-guided BoNT-A injection in spasticity (O.K., M.V-G., R.R.) [42] and an article exploring the role of US in cervical dystonia treatment (T.B.) [3].

### 5.3. Delphi Survey Rounds

#### 5.3.1. Round 1

Round 1 was a face-to-face advisory board meeting, convened in November 2022 and attended by all nine experts. In a series of presentations and discussions, experts explored anatomy and BoNT-A injection training programs in the geographical regions represented by the panel (USA, Canada, Germany, Scandinavia). There was also a practical session in which experts injected different agents into the muscles of cadaveric specimens to simulate BoNT-A injection, both with and without US guidance. This helped guide the experts’ deliberations about how anatomy, muscle localization, and BoNT-A injection techniques should be taught in training courses.

There was no voting in Round 1. Instead, experts generated a set of initial statements describing key best practices for developing training courses for anatomy teaching and BoNT-A injection techniques for spasticity and dystonia treatment. These initial statements were refined in subsequent rounds.

#### 5.3.2. Round 2

In Round 2, conducted in February 2023, the initial 16 statements generated in the advisory board meeting (Round 1) were circulated to experts as an online questionnaire via Typeform. Experts were requested to vote on the statements by marking each with “agree” or “disagree,” and were encouraged to revise any statements they disagreed with. Consensus for any statement was achieved if 80% of experts agreed with it. Statements that achieved consensus were retained without modification, while statements that did not achieve consensus (<80% agreement) were modified according to experts’ feedback and used to develop the questionnaire for Round 3. Experts remained anonymous throughout Round 2.

#### 5.3.3. Round 3

Round 3 was conducted in March/April 2023. After modification, statements that did not reach consensus in Round 2 were circulated to experts in another online questionnaire via Typeform (the original statements were also provided so that experts could reflect on the previous statements). Experts voted in the same way as in Round 2, with consensus again defined as 80% agreement for any given statement and with experts remaining anonymous throughout the voting process. A final list of statements describing best-practice principles for developing training courses for anatomy teaching and BoNT-A injection techniques was generated at the end of Round 3, comprising all statements that achieved consensus and those that did not.

## Figures and Tables

**Table 1:** Final statements after a three-round modified Delphi consensus process.

Statement	Consensus Level
Consensus	
The core learning objectives should be based on target audience and region.	100%
1a. How can we enhance existing educational programs? ^1^	100%
1b. How can we improve knowledge and competency of the target audience? ^1^	100%
2. The target audience is important for the program design (e.g., university-level faculty, residents, or any educator).	100%
2a. Depending on the level of the training (basic or advanced) and limited availability, a recommendation or qualification for participation in trainings should be considered.	100%
2b. Programs should look beyond physiatrists and neurologists as participants.	88%
2c. Depending on local scope of practice, programs can be tailored for allied health colleagues (i.e., physiotherapists, occupational therapists) to enhance their knowledge of anatomy related to post-stroke spasticity.	89%
3. A curriculum with a simple design and an adaptable framework is needed.	100%
4. The training should provide participants with skills for which objective improvement can be demonstrated after the conclusion of the course.	100%
5. Trainers and participants should be able to demonstrate their engagement during the training program through hands-on activities.	100%
5a. Participants should have the opportunity to perform guidance techniques (e.g., ultrasound [US]) in the training.	100%
5b. If possible, trainers should provide trainees with (pre- and) post-training assessments.	89%
6. A program needs to assure uniformity of the teaching approach and consistency in coverage of key topics in the training program across different dates and regions.	100%
6a. Instructors in all trainings should align on the use of similar nomenclature to describe critical procedures and anatomy.	100%
7. All educational programs should strive for diversity, equity, and inclusion of trainers by considering the competencies in the specific areas trained.	89%
8. Participants should have a basic understanding of US in case those basics (like knobology or ergonomics) are not part of the training.	89%
9. The training program should include additional instruction on the importance of other techniques (electromyography [EMG], electrical stimulation [e-stim], computed tomography [CT]) besides US-guided injection to ensure that the participants learn about other techniques.	89%
9a. What are the limitations and pitfalls of the different techniques? ^1^	88%
9b. What evidence supports these techniques? ^1^	88%
10. Botulinum neurotoxin injections represent an important part of a broader holistic multimodal approach to treating movement disorders.	100%
12. A training program should include how to implement goal-setting and how to evaluate patients.	100%
13. The lead organizer/trainer should conduct, if possible, “Train the trainer” meetings (e.g., lunches/dinners) to ensure alignment between all instructors before the training.	100%
14. When designing a program, identify available strengths in local university/hospital departments and find ways to leverage them for teaching.	100%
15. Participants should have the opportunity to complete a post-training survey to rate the instructors in their role as educators.	100%
16. If available, cadaveric specimens (full body or prosection) should be used to enhance anatomy teaching for spasticity management and US-guided techniques.	100%
16a. Virtual models can, to some degree, substitute for actual cadaveric specimens for teaching anatomy.	88%
16b. Artificial models can be used as an additional training tool besides cadaveric specimens for teaching US and injection techniques.	100%
No consensus	
11. As part of a holistic multimodal approach to treat movement disorders, the word “injector” should be replaced with “experts in the management of spasticity and movement disorders”.	78%

^1^ Sub-statements that take the form of open-ended questions indicate issues that should be considered in light of the corresponding primary statement. Specifically, sub-statements 1a and 1b indicate that experts responsible for developing and delivering training programs should consider how to enhance existing educational programs (1a) and how to improve the knowledge and competency of the target audience (1b) when selecting core learning objectives based on target audience and region. Sub-statements 9a and 9b indicate that when experts are deciding which guidance techniques to include in the training program, they should also consider adding instruction about the limitations and pitfalls of the techniques (9a) and the evidence supporting these techniques (9b).

**Table 2:** Advantages of using cadaveric specimens in the development of injection approach: authors’ perspectives and experiences.

Advantages of Using Cadavers	
Aids understanding of muscle anatomy	Cadaver dissection allows for a detailed understanding of muscle anatomy, including the size, shape, and location of individual muscles and their relationship to surrounding structures. This is crucial when targeting specific muscles for injection of BoNT-A.
2. Aids understanding of nerve supply	Cadaver dissection can help clinicians understand the nerve supply to different muscles. This is important for BoNT-A injections, as the toxin must be injected directly into affected muscles to maximize effectiveness. Furthermore, comprehensive understanding of muscle nerve supply allows clinicians to practice diagnostic nerve blocks, which can be an important part of patient assessment. This knowledge also has broader implications for current and developing spasticity treatments, such as chemoneurolysis and cryolysis.
3. Allows practicing injection techniques	Cadavers can provide an opportunity to practice injection techniques in a realistic setting. This can help clinicians improve their skills and become more comfortable with the procedure.
4. Aids understanding of variations	Each human body is unique. By studying cadavers, clinicians can gain a better understanding of the variations that exist in human anatomy, which can help them tailor their injection techniques to individual patients.
5. Allows visualization of deep structures	In some cases, the muscles affected by post-stroke spasticity may be deep within the body. Cadaver dissection can help clinicians visualize these deep structures and understand how to target them with BoNT-A injections.
6. Aids injection safety	Understanding the precise location of muscles, nerves, and blood vessels can help prevent complications, such as injecting the toxin into blood vessels or causing damage to nerves during BoNT-A administration.

## Data Availability

Data are contained within the article or Supplementary Material.

## Supplementary Materials

The following supporting information can be downloaded at: https://www.mdpi.com/article/10.3390/toxins16080371/s1, Table S1: Statements generated at the advisory board meeting (Round 1); Table S2: Statements reaching consensus/no consensus after Round 2 voting.
