# Supplementary material for: Delphi Analysis: Optimizing Anatomy Teaching and Ultrasound Training for Botulinum Neurotoxin Type A Injection in Spasticity and Dystonia
Source: Toxins (Basel). 2024 Aug 21;16(8):371. doi: 10.3390/toxins16080371 (PMC11359033; doi:10.3390/toxins16080371)
Supplement: Supplementary file 1 [file toxins-16-00371-s001.zip › toxins-3025285-supplementary/toxins-3025285-supplementary/Table S2_16Aug24_FINAL.pdf]

Table S2. Statements reaching consensus/no consensus after Round 2 voting.

	Statement	Consensus Level
1.	The core learning objectives should be based on target audience and region.	**100%**
	1a. How can we enhance existing educational programs?[1]	**100%**
	1b. How can we improve knowledge and competency of the target audience?[1]	**100%**
2.	The target audience is important for the program design (e.g., university-level faculty, residents, or any educator).	**100%**
	2a. New participants for training programs should be selected by recommendation.	38%
	2b. Programs should look beyond physiatrists and neurologists as participants.	**88%**
	2c. Also, consider including physical and occupational therapists in the training program.	63%
3.	A curriculum with a simple design and an adaptable framework is needed.	**100%**
4.	The training should provide participants with skills for which objective improvement can be demonstrated after the conclusion of the course.	**100%**
5.	Trainers and participants should be able to demonstrate their engagement during the training program through hands-on activities.	**100%**
	5a. Participants should have the opportunity to perform guidance techniques (e.g., US) in the training.	**100%**
	5b. Trainers should provide pre- and post-training assessments of participants.	63%
6.	A program needs to assure uniformity of the teaching approach and consistency in coverage of key topics in the training program across different dates and regions.	**100%**
	6a. Instructors in all trainings should align on the use of similar nomenclature to describe critical procedures and anatomy.	**100%**
7.	The program needs diversity of the instructors, including the lead trainer.	63%
8.	Participants should understand the basics of US (knobology, ergonomics, needle handling) before attending the training.	25%
9.	The training program should include instruction on the role of multiple guidance techniques (e.g., US, EMG, e-stim) to ensure participants learn as many techniques as possible (not all guidance methods will be available in all clinic settings).	75%
	9a. What are the limitations and pitfalls of the different techniques?[1]	**88%**
	9b. What evidence supports these techniques?[1]	**88%**

10.	Botulinum neurotoxin injections represent an important part of a broader holistic multimodal approach to treating movement disorders.	**100%**
11.	As part of a holistic multimodal approach to treat movement disorders, the word "injector" should be replaced with "experts in the management of movement disorders."	75%
12.	A training program should include how to implement goal-setting and how to evaluate patients.	**100%**
13.	The lead organizer/trainer should conduct, if possible, "Train the trainer" meetings (e.g., lunches/dinners) to ensure alignment between all instructors before the training.	**100%**
14.	When designing a program, identify available strengths in local university/hospital departments and find ways to leverage them for teaching.	**100%**
15.	Participants should have the opportunity to complete a post-training survey to rate the instructors in their role as educators.	**100%**
16.	We need a cadaveric specimen for teaching anatomy, US, and injection techniques.	63%
	16a. Virtual models can, to some degree, substitute for an actual cadaveric specimen for teaching anatomy.	**88%**
	16b. Artificial models can, to some degree, substitute for an actual cadaveric specimen for teaching US and injection techniques.	75%

[1]Sub-statements that take the form of open-ended questions indicate issues that should be considered in light of the corresponding primary statement. Specifically, sub-statements 1a and 1b indicate that experts responsible for developing and delivering training programs should consider how to enhance existing educational programs (1a) and how to improve the knowledge and competency of the target audience (1b) when selecting core learning objectives based on target audience and region. Sub-statements 9a and 9b indicate that when experts are deciding which guidance techniques to include in the training program, they should also consider adding instruction about the limitations and pitfalls of the techniques (9a) and the evidence supporting these techniques (9b).
